# 830. Risk factors for late Pneumocystis jirovecii pneumonia in kidney transplant recipients: A Case-Control Study of United States Renal Data System Data

**DOI:** 10.1093/ofid/ofad500.875

**Published:** 2023-11-27

**Authors:** Maria Alejandra Mendoza, Paschalis Vergidis, Daniel Z P Friedman

**Affiliations:** Mayo Clinic, Rochester, Minnesota; Mayo Clinic, Rochester, Minnesota; University of Chicago, Chicago, Illinois

## Abstract

**Background:**

Organ transplant recipients are at increased risk of potentially life-threatening opportunistic infections, including *Pneumocystis jirovecii* pneumonia (PJP). Given the use of effective prophylaxis, PJP has become less common, especially during the first-year post-transplant. Recent data has shown that the risk of infection has shifted within the first 2 years after discontinuing prophylaxis. We aimed to determine risk factors for late posttransplant PJP using a national database.

**Methods:**

Using the United States Renal Data System database, we performed a retrospective case-control study of patients who underwent kidney transplant from 1998 through 2019. To evaluate risk factors for late PJP, we performed logistic regression analysis by comparing characteristics between infected patients and their matched uninfected controls.

**Results:**

We identified 407 cases with PJP and matched to 1628 controls (1:4). 60 (14.7%) of the cases occurred within one year post transplant and 347 (85.3%) patients were diagnosed after one year of transplant. In the late period, the median time between transplant and PJP diagnosis was 68.7 (IQR 28 -134.2) months. Seventy cases (20.1%) occurred between 12 and 24 months.

In multivariate analysis (table 1), risk factors for late infection included a higher Elixhauser Comorbidity Index (ECI), history of cytomegalovirus disease, older donor age, use of antilymphocyte agents, history of re-transplant and a longer time of hemodialysis prior to transplant. Of note, in the early period, we found that having a living donor was protective.

**Figure:**
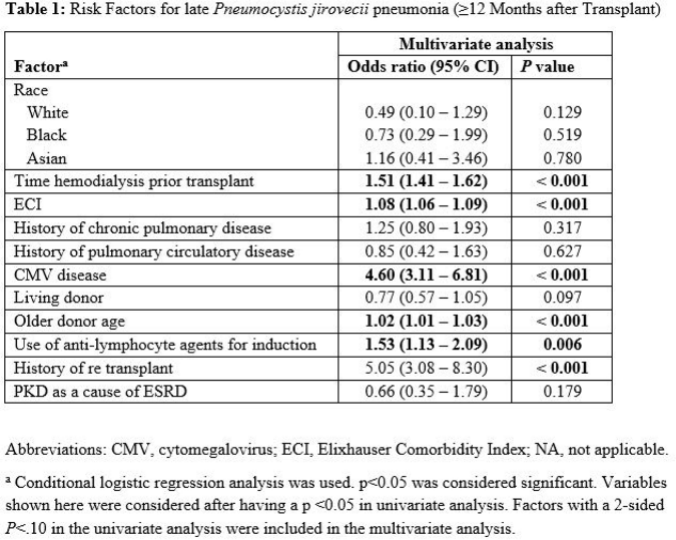

Multivariate analysis of risk factors for late Pneumocystis jirovecii pneumonia

**Conclusion:**

Our findings highlight the importance of a timely transplant to mitigate the risk of post-transplant PJP in the late post-transplant period where prophylaxis is not routinely used. These results also raise the consideration of PJP prophylaxis in the late period in patients diagnosed with CMV disease, history of re transplant and recent use of antilymphocyte agents.

**Disclosures:**

**Paschalis Vergidis, MD, MSc**, AbbVie: Advisor/Consultant|Ansun: Grant/Research Support|Cidara: Grant/Research Support|Scynexis: Grant/Research Support

